# Risk factors for poor treatment outcomes of 2266 multidrug-resistant tuberculosis cases in Ho Chi Minh City: a retrospective study

**DOI:** 10.1186/s12879-020-4887-1

**Published:** 2020-02-22

**Authors:** Le Hong Van, Phan Trieu Phu, Dao Nguyen Vinh, Vo Thanh Son, Nguyen Thi Hanh, Le Thanh Hoang Nhat, Nguyen Huu Lan, Truong Van Vinh, Nguyen Thi Mai Trang, Dang Thi Minh Ha, Guy E. Thwaites, Nguyen Thuy Thuong Thuong

**Affiliations:** 1 Tuberculosis group, Oxford University Clinical Research Unit, 764 Vo Van Kiet street, District 5, Ho Chi Minh City, Vietnam; 2 Pham Ngoc Thach Hospital, Ho Chi Minh City, Viet Nam; 3 Centre for Tropical Medicine and Global Health, Nuffield Department of Medicine, University of Oxford, Oxford, UK

**Keywords:** Multidrug resistant tuberculosis, Retrospective, Treatment outcome, Risk factors, Vietnam

## Abstract

**Background:**

Multidrug resistant tuberculosis (MDR-TB) remains a serious public health problem with poor treatment outcomes. Predictors of poor outcomes vary in different regions. Vietnam is among the top 30 high burden of MDR-TB countries. We describe demographic characteristics and identify risk factors for poor outcome among patients with MDR-TB in Ho Chi Minh City (HCMC), the most populous city in Vietnam.

**Methods:**

This retrospective study included 2266 patients who initiated MDR-TB treatment between 2011 and 2015 in HCMC. Treatment outcomes were available for 2240 patients. Data was collected from standardized paper-based treatment cards and electronic records. A Kruskal Wallis test was used to assess changes in median age and body mass index (BMI) over time, and a Wilcoxon test was used to compare the median BMI of patients with and without diabetes mellitus. Chi squared test was used to compare categorical variables. Multivariate logistic regression with multiple imputation for missing data was used to identify risk factors for poor outcomes. Statistical analysis was performed using R program.

**Results:**

Among 2266 eligible cases, 60.2% had failed on a category I or II treatment regimen, 57.7% were underweight, 30.2% had diabetes mellitus and 9.6% were HIV positive. The notification rate increased 24.7% from 2011 to 2015. The treatment success rate was 73.3%. Risk factors for poor treatment outcome included HIV co-infection (adjusted odds ratio (aOR): 2.94), advanced age (aOR: 1.45 for every increase of 5 years for patients 60 years or older), having history of MDR-TB treatment (aOR: 5.53), sputum smear grade scanty or 1+ (aOR: 1.47), smear grade 2+ or 3+ (aOR: 2.06), low BMI (aOR: 0.83 for every increase of 1 kg/m2 of BMI for patients with BMI < 21).

**Conclusion:**

The number of patients diagnosed with MDR-TB in HCMC increased by almost a quarter between 2011 and 2015. Patients with HIV, high smear grade, malnutrition or a history of previous MDR-TB treatment are at greatest risk of poor treatment outcome.

## Background

Multidrug resistant tuberculosis (MDR-TB), defined as tuberculosis (TB) with resistance to at least rifampicin and isoniazid, is a serious public health problem. In 2017, there were an estimated 558,000 incident cases and 230,000 deaths due to MDR/Rifampicin resistant (RR)-TB worldwide. Treatment of MDR-TB is lengthy, toxic and expensive, with success rates reported globally at 55% in 2017 [1]. HIV co-infection, low body mass index (BMI) and positive sputum smear are predictors of poor MDR-TB outcomes, but the effects of these predictors may vary in different regions [2–6].

Vietnam is listed by World Health Organization as having a high TB and MDR-TB burden. The estimated incidence of TB in 2017 was 129 per 100,000 people. A national survey in 2011 showed resistance to any drug was 32.7% in new TB patients and 54.2% in previously treated patients [7]. The prevalence of MDR-TB was 4.1% in new patients and 17% in previously treated patients, with 4900 estimated new cases annually countrywide [1]. Ho Chi Minh City (HCMC) is the most populous city in Vietnam (around 8 million people) and also the center for TB and drug-resistant TB management in Southern Vietnam. In 2009, the Vietnam National TB Programme initiated the Programmatic Management of Drug-resistant TB (PMDT) under the support of Global Fund (Switzerland) to provide free treatment and support for MDR-TB patients [2]. Pham Ngoc Thach hospital (PNTH) is a tertiary referral center for TB and lung diseases in Southern Vietnam, which provided treatment for 81% of registered MDR-TB patients in Vietnam in 2010 [8]. The number of patients enrolled in PMDT rapidly increased from 2010 to 2014; yet, the total number of MDR-TB patients enrolled for treatment in Vietnam in 2014 was still very low, with only a third of the estimated 5100 cases receiving treatment [2]. From 2011, all 24 district TB units (DTUs) of HCMC participated in the MDR-TB management. However, until now, there has been no information about the notification trend, treatment outcome and factors associated with poor treatment outcome of MDR-TB patients in HCMC. In this study, we retrospectively investigated the demographic characteristics and risk factors for poor treatment outcomes of MDR-TB in HCMC from 2011 to 2015.

## Methods

### Study setting and population

Patients were hospitalized in PNTH for 7 to 14 days to initiate treatment, and then referred to DTUs for outpatient follow-up. Treatment modalities did not change during the study time, with the standardized combination of 6 drugs for a total of 18 to 24 months of treatment [2]. Table A in supplementary data outlines the standardized treatment regimens used in the study time and Table B describes the treatment outcomes of MDR-TB.

Sputum samples from suspected MDR-TB patients or from patients with MDR/RR-TB detected by XpertMTB/RIF or line probe assay (LPA) were sent to PNTH to confirm MDR-TB by phenotypic drug susceptibility testing (DST). Culture and DST in solid and liquid media was the main diagnostic method prior 2012. Although pyrazinamide and ethambutol were part of the standardized MDR-TB regimen, detecting resistance to these drugs was expected to be challenging due to the poor performance of phenotypic DST assays [9]. GenoType MTBDR*plus* (Hain Lifescience GmbH, Germany - LPA) and Xpert MTB/RIF (Cepheid, USA) were introduced to detect MDR/RR-TB in 2010 and 2012 respectively. We later use the term “DST” to refer to phenotypic DST in our results.

**Fig. 1:**
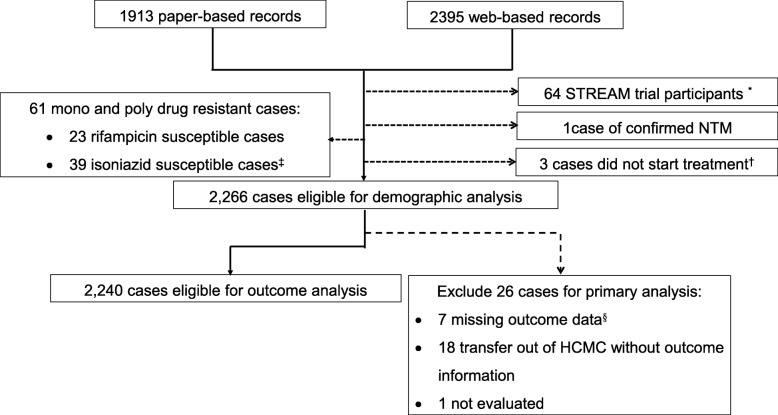
Flow diagram of eligible cases for analysis. * 64 STREAM trial participants from 2012 and 2015 were excluded as they received 9-month regimen and were not enrolled in the PMDT. ^†^ 3 patients died before MDR-TB treatment. ^‡^ 39 isoniazid susceptible TB patients received MDR-TB treatment, including 38 cases with mono rifampicin resistance and 1 case with mono streptomycin resistance (susceptible to both isoniazid and rifampicin). ^§^ Missing outcome data: both treatment outcome and culture results were not recorded

We included all patients who initiated treatment with second-line drugs in HCMC under PMDT from January 2011 to December 2015. To ensure that all treatment outcomes could be captured, more recently diagnosed cases were excluded. Patients were excluded if 1) MDR-TB was diagnosed by a molecular assay but DST reported rifampicin or isoniazid susceptibility; 2) they were enrolled in the STREAM trial [10] to receive a 9-month regimen; 3) they did not start treatment (Fig. 1).

### Data collection

Demographic and clinical information, radiographs, acid-fast bacilli (AFB) staining, DST results, treatment regimens and treatment outcomes were recorded into structured paper forms. To improve reliability, we collected data from both standardized paper-based treatment cards and electronic records and verified data during the data collection, entry and analysis processes.

### Statistical analysis

Data analysis was performed using R program version 3.5.2 [11]. The baseline characteristics were summarized in terms of the number of cases (percentage) for categorical variables, and median with interquartile range (IQR) for continuous variables. We used a Kruskal Wallis test assess changes in median age and BMI over 5 years, and a Wilcoxon test to compare the median BMI of patients with and without diabetes mellitus (DM). Chi squared tests were used to compare categorical variables.

Multivariate logistic regression was used to identify risk factors contributing to poor treatment outcome. Treatment outcome was binary, defined as “success” (cured, completed) or “non-success” (death, failure, lost to follow-up) as in [12]. HIV co-infection, history of previous MDR-TB treatment, AFB smear grade and BMI were included as covariates in the model. Gender, age and DM status were adjusted for as potential confounders. The covariates age and BMI were modelled in a piecewise linear form. For age we used an index variable (age ≤ 60 years old) and a linear pattern for age greater than 60 years old (Figure A). Similarly for BMI, we used an index variable (BMI ≥ 21 kg/m^2^) and a linear pattern for BMI less than 21 kg/m^2^ (Figure B). To minimize potential bias from missing data, we used multiple imputation by chained equation (“mice” package in R [13]) and performed multivariate logistic regression models using both imputed data analysis and complete case analysis.

## Results

### Characteristics of MDR/RR-TB patients

Two thousand three hundred ninety-five electronic records and 1913 paper-based records were available and 2266 MDR/RR-TB cases were included (Fig. 1). Of these patient diagnosed between 2011 and 2015, eight patients relapsed, 13 were re-treated after loss to follow-up, and two were re-treated after treatment failure. Baseline characteristics are presented in Table 1. Median age among the 2266 cases was 43 years (IQR: 33–53 years) and did not change between 2011 and 2015 (*p* = 0.481). A total of 204 patients (9.6% of tested patients) were HIV co-infected; of these, 33 (16.1%) were registered as new TB patients and 21 (10.3%) had extra-pulmonary MDR-TB, including 10 (4.9%) patients with MDR-TB meningitis. MDR-TB meningitis was more common than in patients without HIV co-infection (*p* < 0.001). Eighty four of 204 HIV co-infected patients (41.2%) were established on antiretroviral therapy (ART) before starting MDR-TB treatment. The remaining HIV co-infected patients (58.8%) started ART at least 2 weeks after starting MDR-TB treatment. Among 1815 cases for whom BMI data were available, 57.8% was classified as underweight and 25.1% severely underweight. Median BMI did not change over 5 years (*p* = 0.966). DM status was available for 1189 patients (52.5%), 359 of whom (30.2%) had DM. Median BMI among patients with DM (20.0 kg/m^2^) was higher than among patients without DM (17.8 kg/m^2^) (*p* < 0.001) and HIV co-infection in patients with DM (0.9%) was lower than among patients without DM (9.8%) (*p* < 0.001).

**Table 1:** Characteristics of MDR-TB patients in HCMC from 2011 to 2015

Characteristic	n (%)
Total	2266
Age at diagnosis (years, median (IQR))	43 (33–53)
> 60 years old	197 (8.7%)
18–60 years old	2037 (89.9%)
< 18 years old	32 (1.4%)
Male	1715 (75.7%)
Site of disease	
Pulmonary	2237 (98.7%)
Multi-organ ^a^	37 (1.7%)
Extra pulmonary	66 (3%)
Lymphadenitis	22
Meningitis	21
Pleuritis	8
Bone and vertebral	4
Soft tissue	1
Gastro-intestinal	1
Registration group	
New	128 (5.6%)
Relapse	678 (29.9%)
Failure of regimen 1	512 (22.6%)
Failure of regiment 2	852 (37.6%)
Treatment after lost to follow-up	47 (2.1%)
Transfer	1 (0%)
Other	46 (2%)
Not recorded	2 (0.1%)
Regimen of previous treatment	
I	913 (40.3%)
II	1012 (44.6%)
III	2 (0.1%)
IV	54 (2.4%)
No history of previous treatment	128 (5.6%)
Unclear history	157 (7%)
BMI at diagnosis (kg/m^2^)	*n* = 1815
Median (IQR)	17.86 (15.76–19.96)
Overweight (BMI ≥ 25)	46 (2.5%)
Normal BMI (BMI: 18.5- < 25)	721 (39.7%)
Mild underweight (BMI: 17- < 18.5)	343 (18.9%)
Moderate underweight (BMI: 16- < 17)	250 (13.8%)
Severe underweight (BMI < 16)	456 (25.1%)
HIV positive	204/2136 tested for HIV (9.6%)
Diabetes	359/1189 (30.2%)
Unknown history of diabetes	1077 (47.5%)
Initial Diagnosis Method	
DST	274 (12.1%)
Xpert	1276 (56.3%)
Hain	705 (31.1%)
DST of last treatment episode	7 (0.3%)
Missing data	4 (0.2%)
AFB smear at baseline	
Positive	1748 (77.1%)
< 1+	158 (9%)
1+	895 (51.1%)
2+	357 (20.5%)
3+	275 (15.7%)
Unknown grade	63 (3.7%)
Negative	475 (21%)
Not recorded	43 (1.9%)
Culture at diagnosis	
Positive	1371 (60.5%)
Negative	178 (7.8%)
Non-Tuberculosis Mycobacterium (but Xpert positive)^b^	4 (0.2%)
Contaminated	19 (0.8%)
Not recorded	694 (30.6%)

^a^ involved both pulmonary and extra pulmonary TB

^b^ All four cases of culture positive for *Non-Tuberculosis Mycobacterium* also had GeneXpert detected *Mycobacterium tuberculosis*

### Drug resistance pattern

Table 2 outlines the observed drug resistance patterns. DST results were retrieved for 502 isolates from 490 patients. Ten patients had results for two isolates at different time points, and one patient had results for three isolates. Among the 490 patients with a DST result, 55.0 and 63.1% had resistance to pyrazinamide and ethambutol, respectively. Resistance to fluoroquinolones and injectable agents was seen in 12.7 and 8.1% of isolates, respectively. Among 378 patients with DST to second-line drugs, 63 (16.7%) had pre extensively drug-resistant (XDR) TB and 8 (2.1%) had XDR-TB.

**Table 2:** Frequency of first and second-line drug resistance of MDR-TB in HCMC, 2011–2015

Drug resistance	n/ total tested (%)
Patients with DST result^a^	490
First line drugs	
Pyrazinamide	210/382 (55.0%)
Ethambutol	298/472 (63.1%)
Streptomycin	438/455 (96.3%)
Second-line drugs	
Fluoroquinolones^b^	48/378 (12.7%)
Any injectable agents^c^	31/384 (8.1%)
All injectable agents	9/115 (7.8%)
Cycloserine	2/240 (0.8%)
Ethionamide/Prothionamide	21/223 (9.4%)

^a^ A total of 502 DST of 490 patients were retrievable

^b^ fluoroquinolones include moxifloxacin, levofloxacin, ofloxacin

^c^ injectable agents include kanamycin, amikacin and capreomycin

### MDR-TB trend

**Fig. 2:**
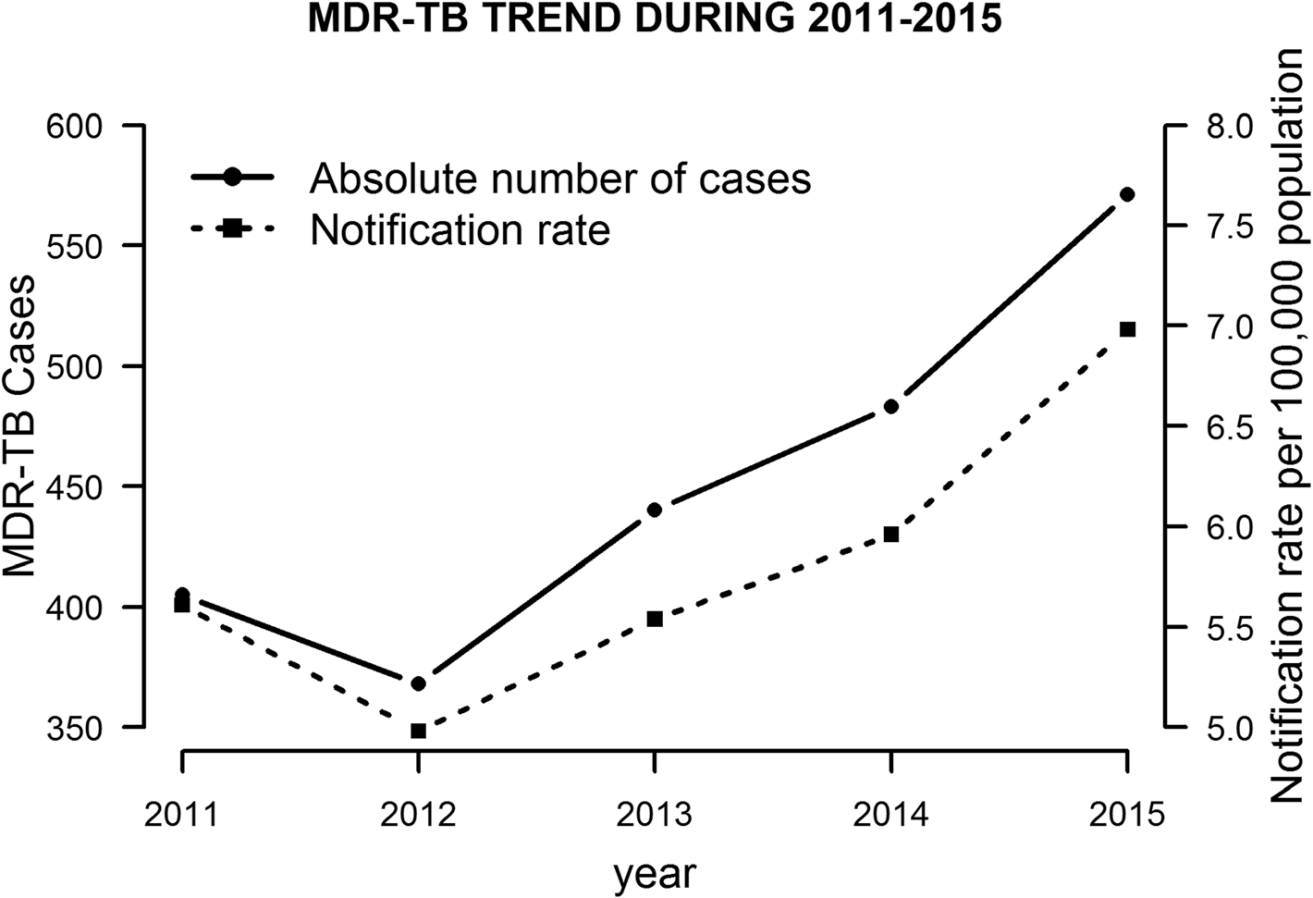
MDR-TB trend for a 5 year period. The absolute number of MDR-TB cases are showned in the solid line, and the notification rate per 100,000 population in the dashed line

Figure 2 shows an increasing temporal trend from 2011 to 2015 for both the absolute number of cases diagnosed and the notification rate per 100,000 population. Numbers of notified MDR/RR-TB patients decreased by 9% between 2011 and 2012, and increased an average of 15.9% annually from 2012 to 2015. The number of MDR-TB cases and the notification rate increased 41.0 and 24.7% from 2011 to 2015, respectively.

### Treatment outcomes

Table 3 summarizes the treatment outcomes of 2240 MDR-TB patients whose treatment outcomes were retrievable. Successful outcomes were achieved in 1641 (73.3%) patients, including 55.6% who were cured and 17.7% who completed treatment but for whom data on cure were unavailable. Among those with unsuccessful outcomes, 10.1% died, 5% failed treatment and 11.6% were lost to follow-up. Patient characteristics by treatment outcome are further described in the supplementary material (Table C). 49/204 patients with HIV died (23.0%), 8 (3.9%) failed treatment and 42 (20.5%) were lost to follow-up. Ten of 21 (47.6%) patients with TB meningitis had successful outcomes, nine (42.6%) died and two (9.5%) were lost to follow-up. Among the 64 patients with pre-XDR-TB, 53.1% had a successful outcome, 14.1% died, 23.4% failed treatment and 7.8% were lost to follow-up. Of 8 XDR-TB patients, 1 (12.5%) was cured with a bedaquiline-containing regimen, 2 (25%) died, including 1 who received a bedaquiline-containing regimen, and 5 (62.5%) failed.

**Table 3:** Treatment outcomes of 2240 MDR-TB patients in HCMC, 2011–2015

Treatment outcome	2011 n (%)	2012 n (%)	2013 n (%)	2014 n (%)	2015 n (%)	Total n (%)
Total (n)	405	365	438	476	556	2240
Cured	246 (60.7%)	190 (52.1%)	225 (51.4%)	289 (60.7%)	296 (53.2%)	1246 (55.6%)
Completed	64 (15.8%)	80 (21.9%)	85 (19.4%)	71 (14.9%)	96 (17.3%)	396 (17.7%)
Died	27 (6.67%)	39 (10.7%)	45 (10.3%)	51 (10.7%)	64 (11.5%)	226 (10.1%)
Failed	19 (4.69%)	20 (5.48%)	23 (5.25%)	22 (4.62%)	29 (5.22%)	113 (5%)
Lost to follow-up	49 (12.1%)	36 (9.86%)	60 (13.7%)	43 (9.03%)	71 (12.8%)	259 (11.6%)

Of 259 patients lost to follow-up, median treatment duration was 200 days (IQR: 60–340) with 56% lost during intensive phase. 17.3% had HIV co-infection, 32% had a positive AFB smear and 35.9% had a positive culture prior to being lost to follow-up.

### Risk factors for poor outcomes

We evaluated the association between poor treatment outcome and HIV co-infection, history of previous MDR-TB treatment, AFB smear grade and BMI. Male gender, age and DM status were included as in the multivariate logistic regression model as potential risk factors. Further analysis failed to show the interaction between HIV co- infection and other risk factors (age, gender, AFB smear grade, BMI, DM status and history of previous MDR-TB treatment) (*p* = 0.93). Since MDR-TB patients received standardized treatment in 24 different DTUs, we did not include treatment site covariate in our final logistic regression model.

**Fig. 3:**
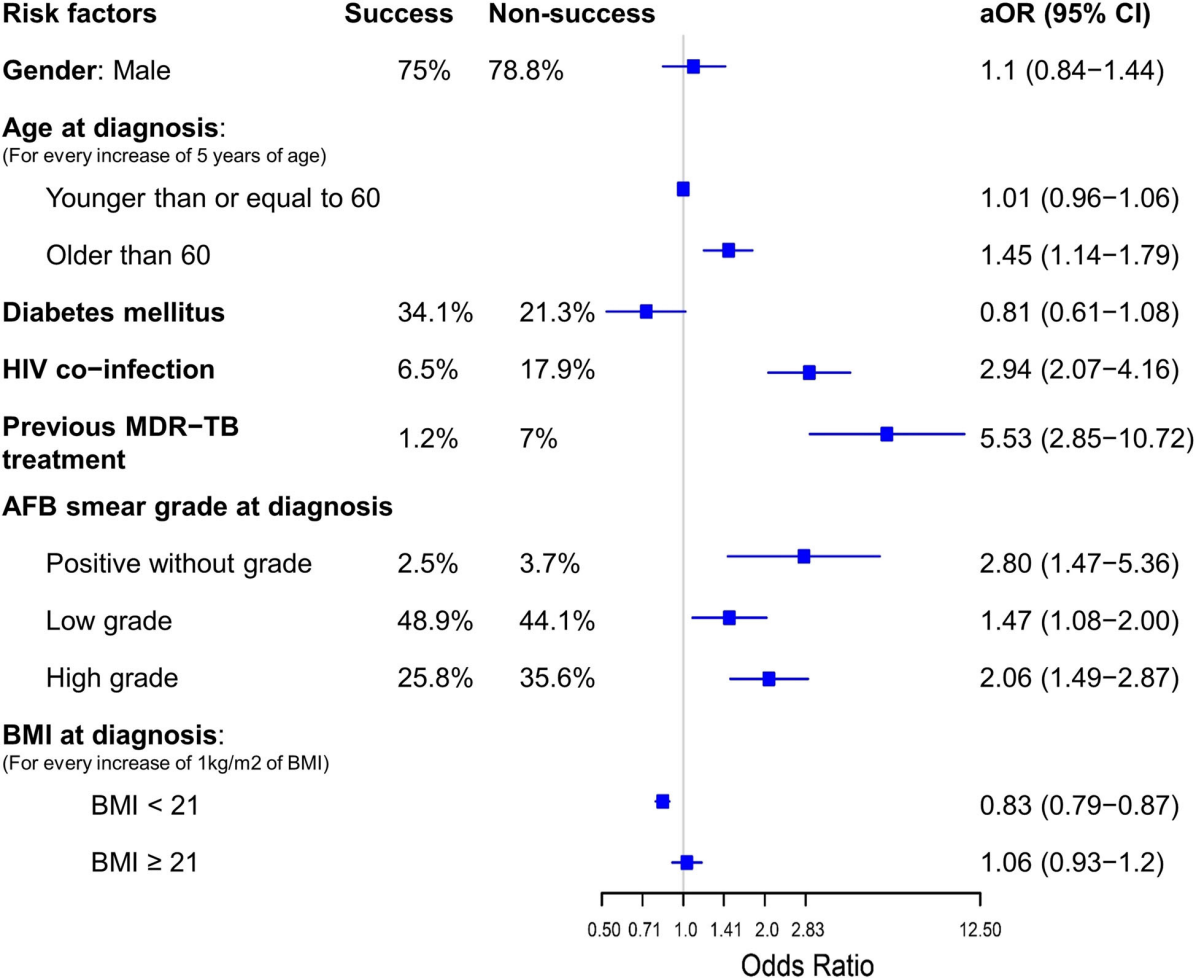
Forrest plot of multivariate multiple imputation logistic regression model showing risk factors for non-success outcome. aOR, adjusted odds ratio; CI, confidence interval; success: the sum of cured and treatment completed

There were only small differences in the results between complete case and multiple imputation analysis (Table 4). Therefore, we presented the Forrest plot of results from the imputed data analysis (Fig. 3).

**Table 4:** Comparison of multivariate logistic regression models using complete case and multiple imputation analysis

Risk factors	Successn (%)	Non-success n (%)	Complete case analysis^a^		Multiple imputation analysis	
			aOR	95% CI	aOR	95% CI
Gender: male	1231 (75.0%)	471 (78.8%)	1.55	0.99–2.42	1.10	0.84–1.44
Age ≤ 60 years			0.98	0.90–1.07	1.01	0.96–1.06
Age > 60 years			1.79	1.26–2.54	1.45	1.14–1.79
	For every increase of 5 years of age					
Diabetes	285 (34.1%)	71 (21.3%)	0.84	0.55–1.31	0.81	0.61–1.08
HIV positive	102 (6.5%)	99 (17.9%)	3.12	1.66–5.84	2.94	2.07–4.16
History of previous MDR-TB treatment	18 (1.2%)	38 (7.0%)	20.37	5.52–75.17	5.53	2.85–10.72
Low smear grade^b^	788 (48.9%)	259 (44.1%)	1.72	1.00–2.95	1.47	1.08–2.00
High smear grade^c^	416 (25.8%)	209 (35.6%)	2.25	1.28–3.93	2.06	1.49–2.87
AFB positive unknown	40 (2.5%)	22 (3.7%)	2.70	1.08–6.78	2.80	1.47–5.36
BMI < 21			0.82	0.76–0.89	0.83	0.79–0.87
BMI ≥ 21			0.96	0.81–1.15	1.06	0.93–1.2
	For every 1 increase of BMI					

^a^ Complete case analysis: non-imputed data

^b^ Low smear grade: scanty or 1+ on AFB smear

^c^ High smear grade: 2+ or 3+ on AFB smear

Independent risk factors for poor outcomes were older age (OR for every increase of 5 years when patients are older than 60: 1.45, 95% CI: 1.14–1.79, *p* < 0.001), HIV co-infection (OR: 2.94, 95% CI: 2.07–4.16, *p* < 0.001), a history of MDR-TB treatment (OR: 5.53, 95% CI: 2.85–10.72, *p* < 0.001), AFB positive (OR: 1.47 for low smear grade (1+ and < 1+), 95%CI: 1.08–2.00, *p* = 0.01 and OR: 2.06 for high smear grade (2+ and 3+), 95%CI: 1.49–2.87, *p* < 0.001), and low BMI (OR: 0.83 for every increase of 1 kg/m2 for patients with BMI < 21, 95%CI: 0.79–0.87, *p* < 0.001) (Fig. 3).

## Discussion

This is the first study to describe the characteristics and identify the risk factors for poor outcomes of MDR-TB in HCMC, Vietnam. Although the incidence of TB in Vietnam has been declining [1], MDR-TB incidence is increasing. There was a shortage of Hain tests in the late 2011 and early 2012, which caused the drop in notified cases (information from annual report of HCMC TB program 2011–2012). The introduction of Xpert at the end of 2012 together with changes in MDR-TB diagnostic policies might have contributed to the increase in notified cases. Transmission of drug-resistant TB may also have contributed. With the rollout of Xpert, all smear positive patients could be screened for drug resistance since the end of 2015. The high rates of failure of regimen 1 (22.6%) and regimen 2 (37.6%) in patients eventually diagnosed with MDR-TB reflect previous inadequate screening for drug resistance among new and retreated patients, and highlight the importance of drug resistance screening for all TB patients regardless of their TB history.

In this RR/MDR-TB cohort, almost all strains were resistant to streptomycin (96.3%), as previously observed in Vietnam [14–16]. This could be explained by the fact that the streptomycin was widely used in regimen 1 and 2 during the intensive phase for both new and retreated patients.

We found high rates of resistance to pyrazinamide (55.0%) and ethambutol (63.1%) in our MDR-TB cohort, as also reported by other studies [17, 18]. This may reflect the fact that the majority of MDR-TB patients (94.3%) already had exposure to first line anti-TB drugs and might have developed resistance to pyrazinamide and ethambutol during previous treatment. This will have limited the effectiveness of the standardized MDR-TB regimen [19] and emphasizes the need for an approved genotypic DST to rapidly detect pyrazinamide resistance.

Resistance rates to fluoroquinolones (12.7%) and injectable agents (8.1%) were comparable to those seen in Vietnam in 2011 [20] but lower than in South Korea [17] and average global rates [1]. Although these drugs are not used in the regimen 1 or 2, the high rates of resistance might be explained by easy access to antibiotics without prescription in Vietnam [21].

HIV co-infection, positive baseline AFB smear, older age and previous treatment with second-line drugs are main risk factors for poor treatment outcomes in our cohort, which were also observed in cohorts in Estonia, Latvia, Philippines, Russia, Peru [4], and Ukraine [3]. Malnutrition was common (57.8%) and a risk factor for poor outcome (OR: 0.81 for every 1 kg/m^2^ increase of BMI). Low BMI might be a consequence of severe disease and low socio-economic status, which are well-known risk factors for poor outcome in TB. PMDT should focus on nutritional support to improve treatment outcomes.

The prevalence of DM in our cohort (30.2%) was double that among patients with TB (13.7%) in Hanoi, Vietnam [22] and was almost 6 times higher than among the general Vietnamese population in 2013 (5.4%) [23]. Although DM is a known risk factor for poor treatment outcome in TB, for developing MDR-TB and for reducing sputum conversion rate during MDR-TB treatment [24–26], it remains controversial whether DM also leads to poor treatment outcome of MDR-TB [24, 27, 28]. After adjustment for other factors, DM was not an independent risk for poor outcomes in our cohort, which agrees with pooled data analysis from cohorts in Latvia, Korea and Italy [29]. Due to the unavailability of DM treatment information, we do not know whether the effect of DM on MDR-TB treatment was influenced by the use of metformin, a hypoglycemic agent that might improve TB treatment outcomes [30, 31]. As markers of glycemic control among patients with MDR-TB and DM were unavailable, the effect on treatment outcome could not be assessed. Despite these limitations, DM is a common but neglected comorbidity in MDR-TB patients and should be screened for prior MDR-TB treatment.

This study has several limitations. This is a retrospective study and some records were irretrievable at the study time. Demographic information and records of smear, culture and DST was not completely recorded on the electronic database. The majority of patients (78.4%) did not have DST results, and we could not include drug resistance information into multivariate logistic regression models. Finally, the information on smoking and alcohol use were not available in our cohort, although they are known risk factors for poor outcome [32] [33]. Therefore, a prospective study is necessary to provide a comprehensive assessment of risk factors for poor treatment outcome in MDR-TB.

## Conclusion

Despite these limitations, the present study emphasizes the increasing trend of MDR-TB in HCMC between 2011 and 2015 and the need for drug resistance screening for all TB patients. Patients with HIV, high smear grade, malnutrition and history of previous MDR-TB treatment are at high risk of poor outcomes.

## Supplementary information

**Additional file 1.** Details on treatment regimens and outcomes of MDR-TB patients.

**Additional file 2.** Dataset of 2266 MDR-TB patients.

**Additional file 3.** Dataset of drug-susceptibility testing results.

## Data Availability

All data generated or analysed during this study are included in this published article and its supplementary information files in demo.csv and dst.csv.

## Abbreviations

AFB: Acid-fast bacilli
BMI: Body mass index
DM: Diabetes mellitus
DST: Drug susceptibility testing
DTU: District tuberculosis unit
HCMC: Ho Chi Minh City
IQR: Interquartile range
LPA: Line probe assay
MDR: Multidrug resistant tuberculosis
OR: Odds ratio
PMDT: Programmatic Management of Drug-resistant Tuberculosis
PNTH: Pham Ngoc Thach Hospital
RR: Rifampicin resistant
TB: Tuberculosis
XDR: Extensively drug resistance
