# Supplementary material for: Risk factors for poor treatment outcomes of 2266 multidrug-resistant tuberculosis cases in Ho Chi Minh City: a retrospective study
Source: BMC Infect Dis. 2020 Feb 22;20:164. doi: 10.1186/s12879-020-4887-1 (PMC7036193; doi:10.1186/s12879-020-4887-1)
Supplement: Supplementary file 1 — Additional file 1. Details on treatment regimens and outcomes of MDR-TB patients. [file 12879_2020_4887_MOESM1_ESM.docx]

**SUPPLEMENTARY DATA**


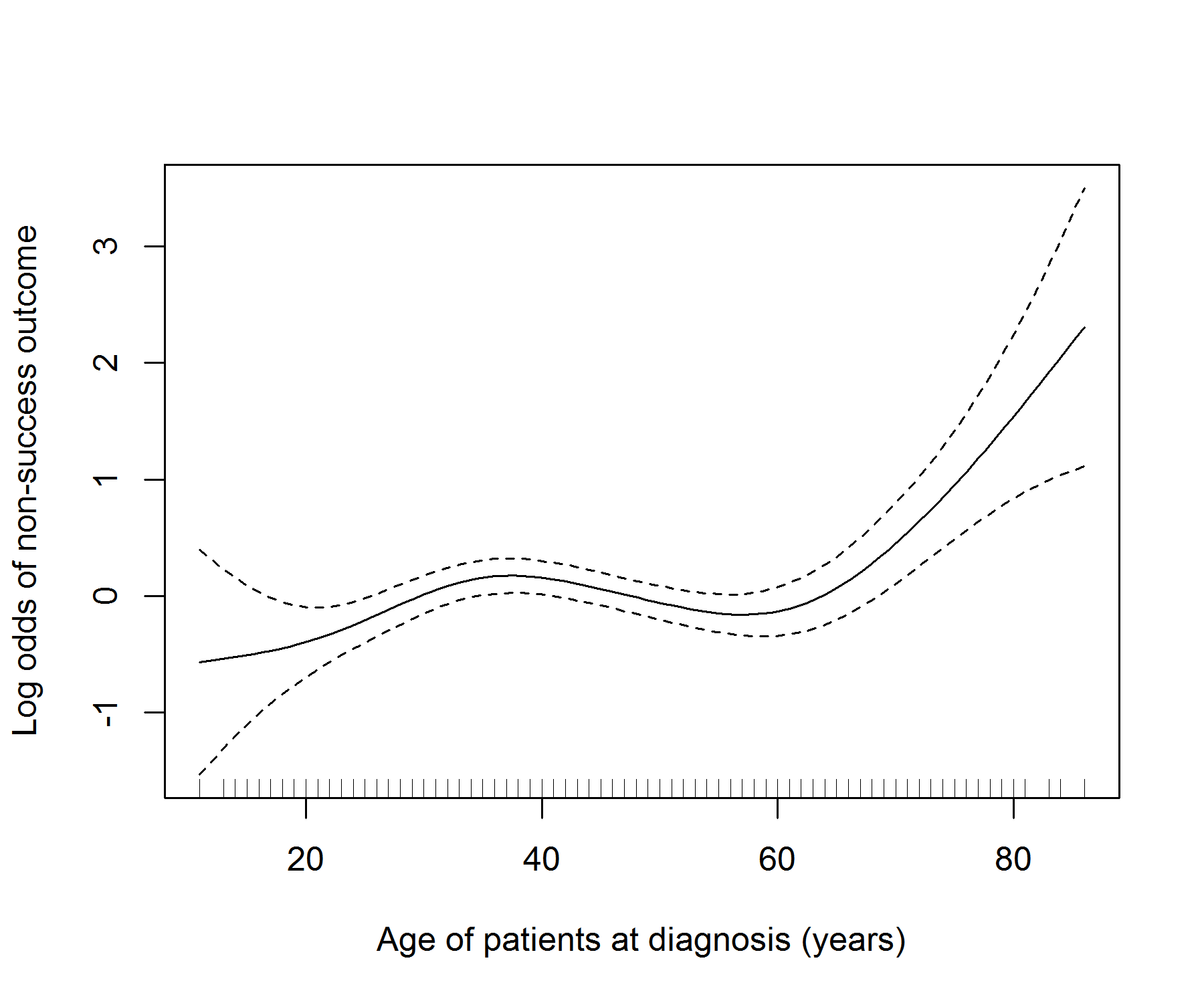


**Figure A:** Generalized Addictive Model of effect of age on treatment outcome


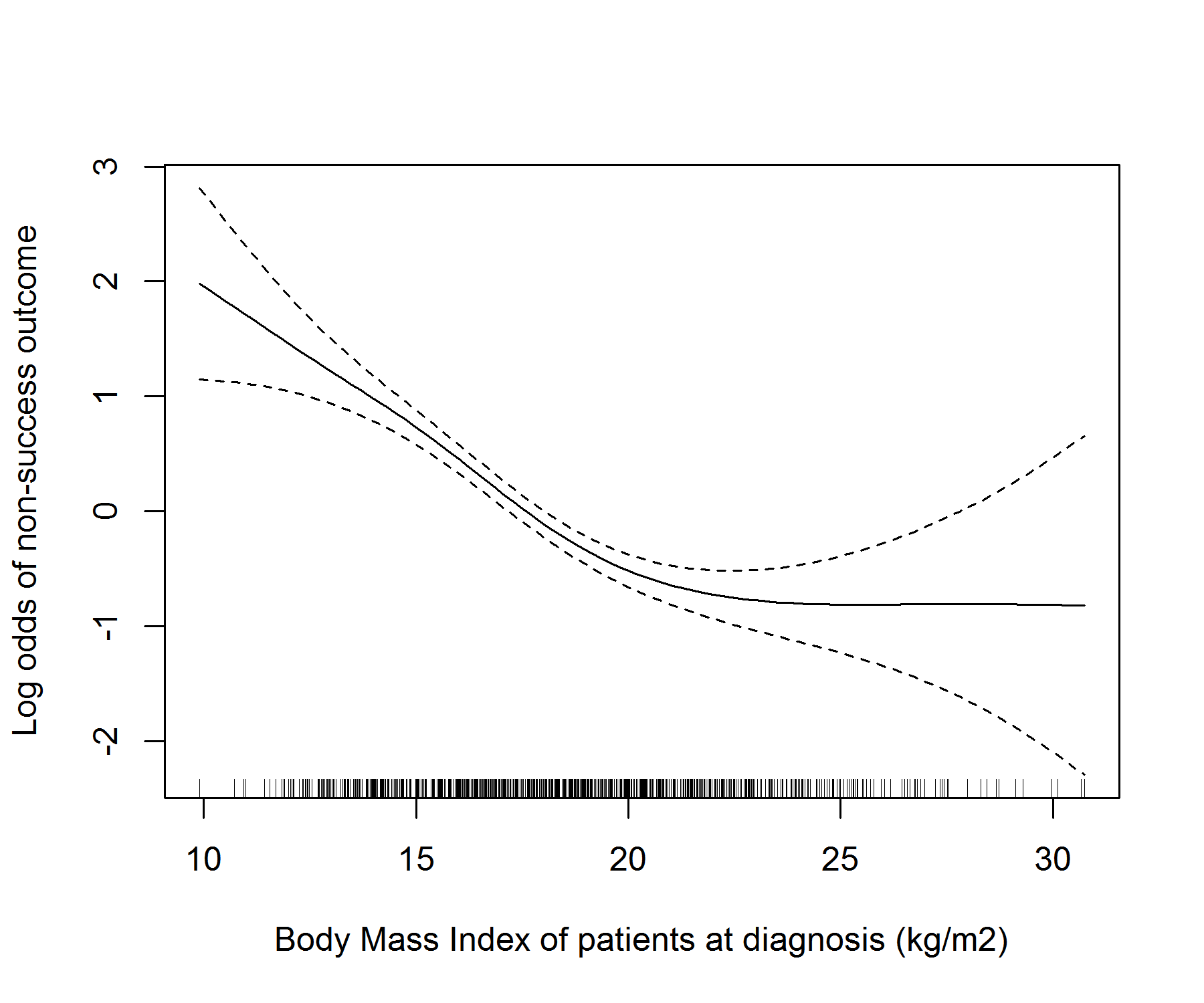


**Figure B:** Generalized Addictive Model of effect of BMI on treatment outcome

**Table A:** Standardized treatment regimens of Vietnam National TB program during 2011-2015

| Regimen | Indication |  |
| --- | --- | --- |
| IA | New adult patients | 2HRZE(S)/4RHE |
| IB | New children patients | 2HRZE/4RH |
| II | Previously treated patients | 2HRZES/1HRZE/5HRE  Or 2SRHZE/1RHZE/5H_3_H_3_E_3_ |
| IIIA | Adult patients with TB of the central nervous system, bone or joint | 2RHZE/10RHE |
| IIIB | Children patients with TB of the central nervous system, bone or joint | 2RHZE/10RH |
| IV | MDR-TB patients | 8Z E Km (Cm) Lfx Pto Cs (PAS)/12Z E Lfx Pto Cs (PAS) |

**Table B:** Definitions of MDR-TB treatment outcomes according to World Health Organization

| Outcome | Definition |
| --- | --- |
| Cured | Treatment completed as recommended by the national guidelines without evidence of failure AND three or more consecutive cultures taken at least 30 days apart are negative after the intensive phase |
| Treatment completed | Treatment completed as recommended by the national guidelines without evidence of failure BUT no record that three or more consecutive cultures taken at least 30 days apart are negative after the intensive phase |
| Failed | Treatment terminated or need for permanent regimen change of at least two anti-TB drugs because of:   - Lack of conversion by the end of the intensive phase; or - Bacteriological reversion in the continuation phase after conversion to negative; or - Evidence of additional acquired resistance to fluoroquinolones or second-line injectable drugs; or - Adverse drug reactions. |
| Died | A patient who dies for any reason during the course of treatment. |
| Lost to follow-up | A patients whose treatment was interrupted for two consecutive months or more. |
| Not evaluated | A patient for whom no treatment outcome is assigned. (This includes cases “transferred out” to another treatment unit and whose treatment outcome is unknown). |
| Treatment success | The sum of Cured and treatment completed. |

**Table C:** Characteristics of patients with success and non-success treatment outcome

| Characteristic | Success  n (%) | Non-success  n (%) |
| --- | --- | --- |
| Total | 1642 | 598 |
| Age at diagnosis (years, median (IQR)) | 43 (32.5-53.5) | 43 (34-52) |
| $\geq$ 60 years old | 130 (7.9%) | 66 (11%) |
| 18-60 years old | 1,488 (90.6%) | 525 (87.8%) |
| $\leq$18 years old | 24 (1.5%) | 7 (1.2%) |
| Male | 1,231 (75.0%) | 471 (78.8%) |
| Site of disease |  |  |
| Pulmonary | 1,626 (99%) | 590 (98.7%) |
| Extra pulmonary | 41 (2.5%) | 19 (3.2%) |
| Multi-organ | 25 (1.5%) | 11 (1.8%) |
| Registration group |  |  |
| New | 79 (4.8%) | 46 (7.7%) |
| Relapse | 512 (31.2%) | 156 (26.1%) |
| Failure of regimen 1 | 403 (24.5%) | 104 (17.4%) |
| Failure of regiment 2 | 613 (37.3%) | 233 (39%) |
| Treatment after lost to follow-up | 16 (1%) | 31 (5.2%) |
| Transfer | 1 (0.1%) | 0 (0%) |
| Other | 18 (1.1%) | 28 (4.7%) |
| BMI at diagnosis (kg/m^2^) | 18.36 (16.34-20.38) | 16.44 (14.72-18.16) |
| HIV positive | 102/1558 tested for HIV (6.5%) | 99/554 tested for HIV (17.87%) |
| Diabetes | 285/837 (34.1%) | 71/334 (21.3%) |
| Unknown history of diabetes | 805 (49.0%) | 264 (44.1%) |
| AFB smear at baseline |  |  |
| Positive | 1,244 (75.8%) | 490 (81.9%) |
| < 1+ | 126 (10.1%) | 31 (6.3%) |
| 1+ | 662 (53.2%) | 228 (46.5%) |
| 2+ | 249 (20%) | 104 (21.2%) |
| 3+ | 167 (13.4%) | 105 (21.4%) |
| Unknown grade | 40 (3.2%) | 22 (4.5%) |
| Negative | 369 (22.5%) | 97 (16.2%) |
| Not recorded | 29(1.8%) | 11 (1.8%) |
| Culture at diagnosis |  |  |
| Positive | 1,017 (61.9%) | 339 (56.7%) |
| Negative | 139 (8.5%) | 39 (6.5%) |
| Non-Tuberculosis Mycobacterium (but Xpert positive) | 3 (0.2%) | 1 (0.2%) |
| Contaminated | 16 (1%) | 3 (0.5%) |
| Not recorded | 173 (10.5%) | 75 (12.5%) |
